# VCAM-1 Targeted Lipopolyplexes as Vehicles for Efficient Delivery of shRNA-Runx2 to Osteoblast-Differentiated Valvular Interstitial Cells; Implications in Calcific Valve Disease Treatment

**DOI:** 10.3390/ijms23073824

**Published:** 2022-03-30

**Authors:** Geanina Voicu, Daniela Rebleanu, Cristina Ana Mocanu, Gabriela Tanko, Ionel Droc, Cristina Mariana Uritu, Mariana Pinteala, Ileana Manduteanu, Maya Simionescu, Manuela Calin

**Affiliations:** 1 “Medical and Pharmaceutical Bionanotechnologies” Laboratory, Institute of Cellular Biology and Pathology “Nicolae Simionescu” of the Romanian Academy, 050568 Bucharest, Romania; geanina.voicu@icbp.ro (G.V.); daniela.rebleanu@icbp.ro (D.R.); cristina.mocanu@icbp.ro (C.A.M.); ileana.manduteanu@icbp.ro (I.M.); maya.simionescu@icbp.ro (M.S.); 2 “Pathophysiology and Pharmacology” Department, Institute of Cellular Biology and Pathology “Nicolae Simionescu” of the Romanian Academy, 050568 Bucharest, Romania; gabriela.tanko@icbp.ro; 3 Central Military Hospital “Dr. Carol Davila”, Cardiovascular Surgery Clinic, 010825 Bucharest, Romania; ionel.droc@gmail.com; 4 Centre of Advanced Research in Bionanoconjugates and Biopolymers, “Petru Poni” Institute of Macromolecular Chemistry, 700487 Iasi, Romania; cristina-mariana.uritu@umfiasi.ro (C.M.U.); pinteala@icmpp.ro (M.P.); 5 Advanced Centre for Research-Development in Experimental Medicine, Grigore T. Popa University of Medicine and Pharmacy of Iasi, 700115 Iasi, Romania

**Keywords:** calcific aortic valve disease, lipopolyplexes, Runx2, shRNA, valvular interstitial cells, VCAM-1

## Abstract

Calcific aortic valve disease (CAVD) is a progressive inflammatory disorder characterized by extracellular matrix remodeling and valvular interstitial cells (VIC) osteodifferentiation leading to valve leaflets calcification and impairment movement. Runx2, the master transcription factor involved in VIC osteodifferentiation, modulates the expression of other osteogenic molecules. Previously, we have demonstrated that the osteoblastic phenotypic shift of cultured VIC is impeded by Runx2 silencing using fullerene (C60)-polyethyleneimine (PEI)/short hairpin (sh)RNA-Runx2 (shRunx2) polyplexes. Since the use of polyplexes for in vivo delivery is limited by their instability in the plasma and the non-specific tissue interactions, we designed and obtained targeted, lipid-enveloped polyplexes (lipopolyplexes) suitable for (1) systemic administration and (2) targeted delivery of shRunx2 to osteoblast-differentiated VIC (oVIC). Vascular cell adhesion molecule (VCAM)-1 expressed on the surface of oVIC was used as a target, and a peptide with high affinity for VCAM-1 was coupled to the surface of lipopolyplexes encapsulating C60-PEI/shRunx2 (V-LPP/shRunx2). We report here that V-LPP/shRunx2 lipopolyplexes are cyto- and hemo-compatible and specifically taken up by oVIC. These lipopolyplexes are functional as they downregulate the Runx2 gene and protein expression, and their uptake leads to a significant decrease in the expression of osteogenic molecules (OSP, BSP, BMP-2). These results identify V-LPP/shRunx2 as a new, appropriately directed vehicle that could be instrumental in developing novel strategies for blocking the progression of CAVD using a targeted nanomedicine approach.

## 1. Introduction

Calcific aortic valve disease (CAVD) is the most common heart valve disorder with increased prevalence in people over 65 years [1,2]. The inflammation plays a crucial role in the onset and progression of CAVD, which starts with valvular endothelial cells (VEC) inflammation and dysfunction that contribute to the immune cell recruitment in the subendothelial space, and the creation of a microenvironment that favors activation and osteodifferentiation of valvular interstitial cells (VIC). The latter, actively contributes to aortic valve fibrosis and calcification. Calcification of the aortic valve impairs the valvular motion and impedes the blood flux towards the aorta, leading to cardiac hypertrophy and ultimately, heart failure [2,3]. There are no efficient pharmacological therapies to prevent or reverse CAVD [4], the only effective interventions being surgical aortic valve replacement or the minimally invasive transcatheter aortic valve implantation (TAVI).

Lately, RNA interference (RNAi) emerged as a powerful strategy in gene silencing for therapeutic purposes. The first small interfering (si)RNA-based therapy was approved by the Food and Drug Administration (FDA) in 2019 for the treatment of polyneuropathy in people with hereditary transthyretin (TTR)-mediated amyloidosis. It is commercialized under the name Onpattro^®^ (patisiran), and consists of a liposome formulation of siRNA designed to target a sequence of TTR mRNA and deliver it to the liver (https://www.onpattro.com/, accessed on 25 March 2022). The liposomes comprise ionizable cationic lipids (DLin-MC3-DMA, (6Z,9Z,28Z,31Z)-Heptatriaconta-6,9,28,31-tetraen-19-yl 4-(dimethylamino) butanoate), phospholipid (DSPC, 1,2-distearoyl-sn-glycero-3-phosphocholine), cholesterol, and polyethylene glycol-modified lipids (PEG2000-C-DMG, 1,2-dimyristoyl-rac-glycero-3-methoxypolyethylene glycol-2000). Once injected into the blood, the lipid nanoparticles are opsonized by apolipoprotein E (ApoE), bind to ApoE receptors on hepatocytes, and are internalized by endocytosis. Following endocytosis, the ionization of the lipid component takes place, a fact favoring the fusion between the liposome and endosomal membranes and the release of the entrapped siRNA into the cytoplasm. The endogenous RNAi mechanism of the cells is employed to process siRNA before binding to the target messenger RNA and degrading it [5]. For cardiovascular disease (CVD) treatment, some encouraging results using RNAi were obtained in animal models, highlighting its high potential as a novel therapy for CVD [6].

Currently, one medication, namely Leqvio^®^ (inclisiran), was approved by the European Medicines Agency (EMA) to lower cholesterol blood levels in people with hypercholesterolemia or mixed dyslipidemia [7]. Inclisiran is a synthetic, small interfering (si)RNA directed against proprotein convertase subtilisin/kexin type 9 (PCSK9) mRNA, conjugated to triantennary N-acetylgalactosamine carbohydrates to ensure a specific binding to asialoglycoprotein receptors expressed on hepatocytes.

It was reported that the osteodifferentiation of VIC is orchestrated by Runt-related transcription factor 2 (Runx2)/Core-binding factor 1 (Cbfa1) that up-regulates the transcription of osteogenic genes such as alkaline phosphatase (ALP), osteopontin (OSP), bone sialoprotein (BSP), or bone morphogenic protein 4 (BMP4) [8]. Thus, we hypothesized that the silencing of Runx2 using RNAi will interfere with the osteoblastic phenotypic shift of VIC and the progress of CAVD. In a previous study, we reported that fullerene (C60)-polyethyleneimine (PEI)/short hairpin (sh)RNA-Runx2 nano-polyplexes efficiently down-regulate Runx2 mRNA and protein expression, and subsequently, significantly reduce the expression of osteogenic proteins (i.e., ALP, BSP, OSP, and BMP4) in osteoblast-differentiated VIC [9].

In this study, we aimed at developing a nanocarrier suitable for in vivo administration and able to perform targeted delivery of shRNA-Runx2 (shRunx2) to affected VIC.

When administered in vivo, polyplexes, due to the overall positive charge, readily interact with plasmatic proteins and accumulate within a few minutes in the reticuloendothelial organs such as the liver or spleen [10]. Thus, strategies to shield polyplexes against non-specific interaction once injected in vivo and direct them to specific cells or tissues are required. It has been reported that lipopolyplexes, namely lipid-coated polyplexes, exhibit superior colloidal stability, reduced cytotoxicity, and higher gene transfection efficiency as compared to polyplexes [11,12,13].

Therefore, we envisioned the development of liposome-encapsulated preformed C60-PEI/shRunx2 polyplexes (lipopolyplexes) that can be functionalized with a suitable ligand to allow specific cellular delivery and increased transfection efficiency. The vascular cell adhesion molecule (VCAM)-1, a transmembrane sialoglycoprotein, was intensively used for targeted drug delivery to endothelium owing to its inducible expression on the cell’s surface in pathological conditions [14,15,16]. Moreover, there is an increased expression of VCAM-1 in aortic VIC after exposure to IFN-γ and LPS [4] or high molecular weight Poly (I: C), a dsRNA mimic [17]. In addition, VCAM-1 is highly expressed in the aortic valve of diabetic/atherosclerotic ApoE-deficient mice and thus, it is an appropriate target for nanocarriers developed to block the progression of CAVD [18].

We report herein the design, preparation, and characterization of lipopolyplexes (LPP) functionalized with a peptide recognizing VCAM-1 and encapsulating C60-PEI/shRunx2 polyplexes (V-LPP/shRunx2) and validation of their functionality in reducing the osteogenic differentiation of aortic valve interstitial cells.

## 2. Results

### 2.1. Design and Characterization of VCAM-1 Targeted Lipopolyplexes

**Figure 1:**
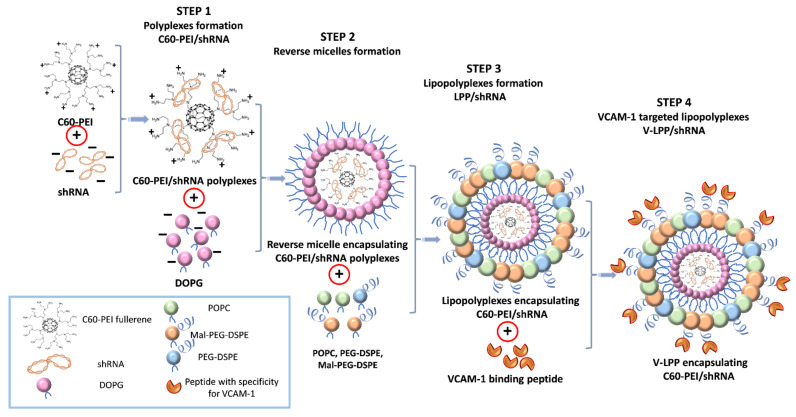
Schematic representation of the successive steps in the synthesis of VCAM-1 targeted lipopolyplexes encapsulating the shRNA plasmid lipopolyplexes (V-LPP/shRNA). First, the core-shell structures consisting of fullerene (C60) core and branched low molecular weight polyethyleneimine (PEI) (2kDa) were complexed with the plasmid containing shRNA sequences specific for Runx2 (shRunx2) or scrambled sequences (shCTR). Second, the anionic phospholipid DOPG dissolved in chloroform/methanol was added to the positively charged C60-PEI/shRNA polyplexes to form reverse micelles entrapping the polyplexes and, third, the organic phase was removed by reverse-phase evaporation under reduced pressure in the presence of coating phospholipids (POPC, Metoxi-PEG2000-DSPE, and Mal-PEG2000-DSPE). The resulting lipopolyplexes, namely PEG-stabilized lipid-coated particles containing the C60-PEI/shRNA polyplexes inside were subsequently extruded through polycarbonate membranes to achieve a narrow and unimodal size distribution of the lipopolyplexes suspension. Next, the VCAM-1 recognizing peptide having an amino acid sequence terminating in cysteine was coupled via a reaction between thiol and maleimide-derivatized PEGylated phospholipid (Mal-PEG2000-DSPE) from the liposome’s membranes.

The VCAM-1 targeted lipopolyplexes consisting of PEG-stabilized liposomes containing the C60-PEI/shRNA polyplexes inside and coupled with VCAM-1 recognizing peptide were obtained by the procedure schematically presented in Figure 1.

The HPLC data indicated an amount of 6.5 μg peptide per μmol lipid coupled to the surface of liposome-encapsulated polyplexes. The quantification of fluorescence of shRNA plasmid encapsulated into LPP using Quant-iT PicoGreen reagent revealed ~9.8 µg shRNA plasmid DNA entrapped per µmol lipid in the lipid-coated particles (98% encapsulation efficiency).

**Figure 2:**
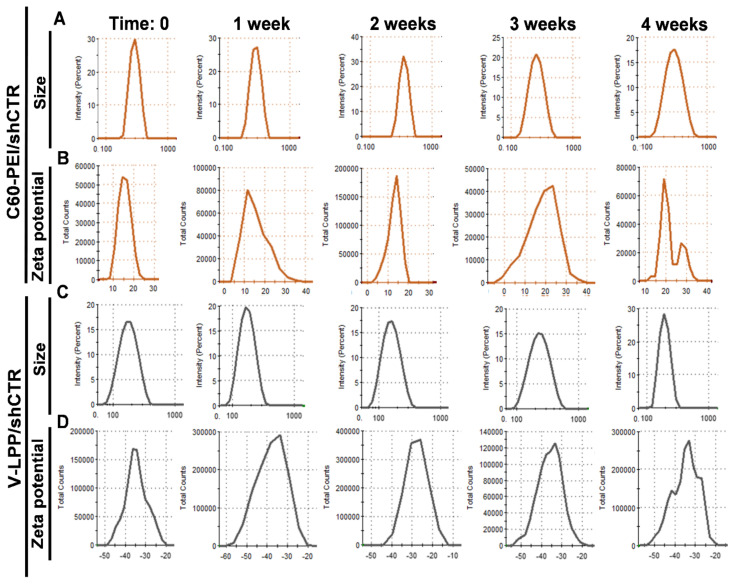
Particle size intensity distribution of C60-PEI/shCTR (**A**) and V-LPP/shCTR (**C**) as measured by dynamic light scattering and Zeta potential distribution of C60-PEI/shCTR (**B**) and V-LPP/shCTR (**D**) measured by electrophoretic light scattering at the initial time and after storage at 4 °C for up to one month.

DLS measurements indicated an average hydrodynamic diameter of ~190 nm for V-LPP/shCTR (Table 1, Figure 2C), a figure that is in good agreement with TEM observations. The lipopolyplexes population was homogenous, as reflected by the heterogeneity polydispersity index (PDI) of ~0.2 [19]. The decreased hydrodynamic diameter of the LPP, compared to the naked C60-PEI/shCTR polyplexes (~270 nm) (Table 1, Figure 2A), indicates the organization of the lipid bilayer structure around the C60-PEI/shCTR core, leading to more compact nanoparticles.

Zeta potential measurements showed that the positive charge of the C60-PEI/shCTR polyplexes at an N/P ratio of 25 (+15 mV) was completely enveloped by the lipid coat, resulting in stable negatively charged nanoparticles (~−30 mV) (Table 1, Figure 2B,D). To examine the stability of lipopolyplexes in time, we measured their size for intervals up to 4 weeks of storage at 4 °C. The data showed no significant changes in the size and ζ-potential of lipopolyplexes V-LPP/shCTR, which are relatively stable within one month compared to polyplexes C60-PEI/shCTR that showed a gradual increase in their dimension with time (Table 1).

**Figure 3:**
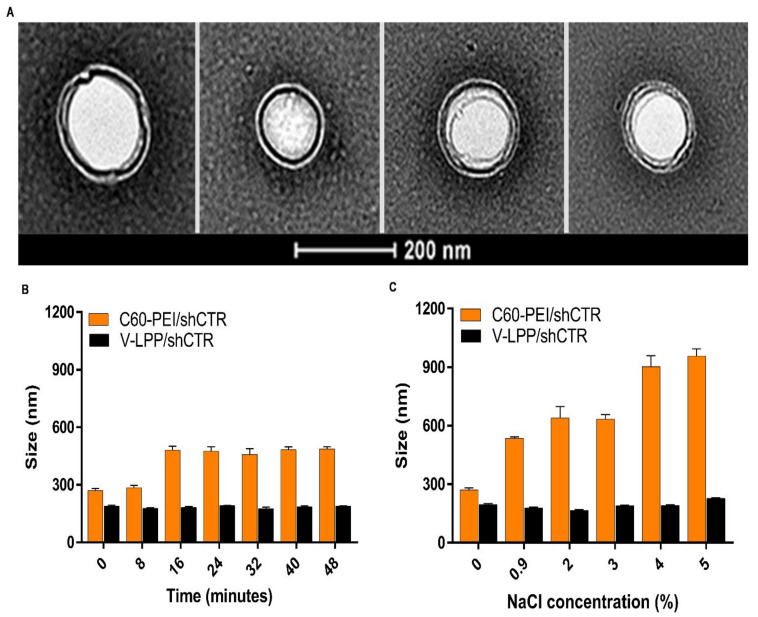
Characterization of VCAM-1 targeted lipopolyplexes encapsulating the shRNA plasmid. (**A**) Electron micrographs of negatively-stained V-LPP/shCTR lipopolyplexes showing its spherical morphology. (**B**) Colloidal stability of C60-PEI/shCTR polyplexes and V-LPP/shCTR lipopolyplexes in PBS determined by DLS. (**C**) Electrolyte-induced flocculation of C60-PEI/shCTR polyplexes and V-LPP/shCTR lipopolyplexes. Note the stability of V-LPP/shCTR lipopolyplexes in comparison with C60-PEI/shCTR polyplexes. Results are reported as mean ± S.D. for three individual measurements.

Negative-staining transmission electron microscopy (TEM) revealed that V-LPP/shCTR lipopolyplexes appear as round structures surrounded by a lipid coat, with uniform size distribution having ~200 nm diameter (Figure 3A).

The colloidal stability of polyplexes and lipopolyplexes was assessed by measuring their size after incubation in PBS for different time intervals (Figure 3B). The mean hydrodynamic diameter of C60-PEI/shCTR polyplexes increased by ~50% after 16 min of incubation in PBS. By comparison, the size of lipid-coated polyplexes (V-LPP/shCTR) was relatively unchanged at the end of the incubation period.

The electrolyte-induced flocculation study performed by the exposure of lipopolyplexes and polyplexes to different sodium chloride concentrations showed that the lipopolyplexes maintained their particle size of ~200 nm for all NaCl concentrations investigated (up to 5%) (Figure 3C). By comparison, for the non-encapsulated polyplexes, a gradual increase in particle size was detected starting at 0.9% NaC_l_, when the size was doubled compared with that measured in the absence of the electrolyte, and reached a value of ~1µm for concentrations above 4% NaC_l_.

### 2.2. Uptake of VCAM-1 Targeted Lipopolyplexes by Valvular Interstitial Cells (VIC)

#### 2.2.1. VCAM-1 Expression on the VIC Surface

The VIC activation and osteodifferentiation were induced by exposing the cells to the culture medium containing 25 mM glucose and osteogenic factors (HGOM) as previously reported [9].

**Figure 4:**
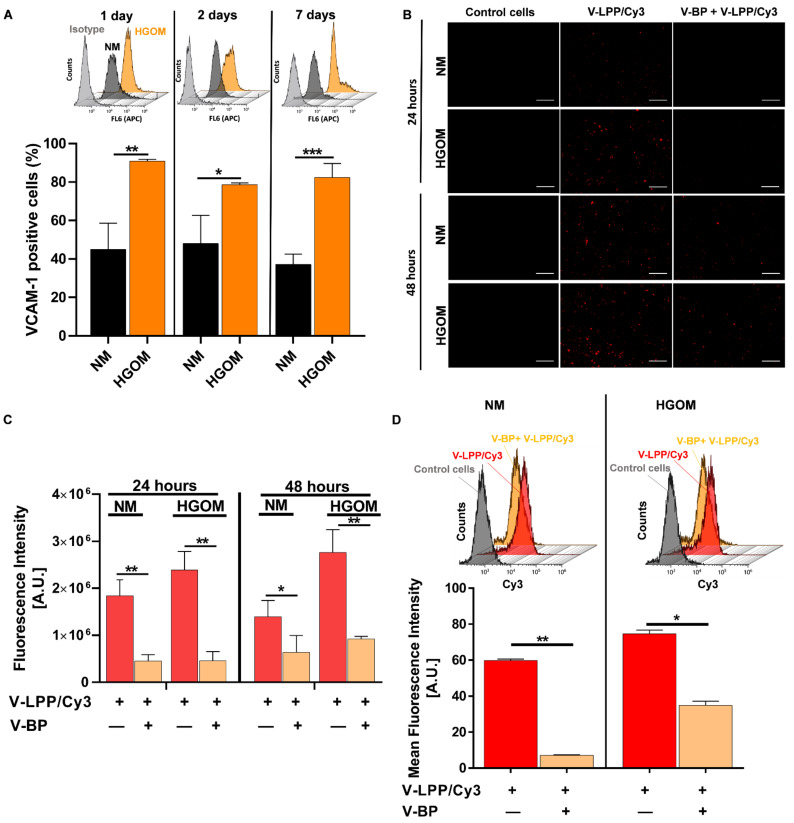
(**A**) VCAM-1 expression on VIC surface. Percentages of VCAM-1 positive cells as determined by flow cytometry experiments on VIC cells cultured in normal medium (NM) or high glucose osteogenic medium (HGOM) for 1, 2, or 7 days and plotted from a representative experiment performed in duplicate. The bar graph shows data as mean ± S.D. The overlays of representative flow cytometry histograms are presented above the graphs. (**B**) Fluorescence microscopy exemplifying the uptake of V-LPP/Cy3 labeled plasmid by VIC exposed to NM or HGOM for 5 days before incubation with lipopolyplexes for 24 and 48 h in the absence or the presence of an excess of V-BP (V-LPP/Cy3 (red); scale bar: 100 μm). (**C**) Quantification of lipopolyplexes uptake expressed as fluorescence intensity of red pixels percentage for each image field (each point represents media of 6 fields) using ImageJ software. The bar graph shows results as mean ± S.D. (**D**) Flow cytometry data showing the uptake of V-LPP/Cy3 labeled plasmid by VIC exposed to NM or HGOM for 5 days before incubation with lipopolyplexes for 48 h in the absence or the presence of excess V-BP. The results are determined as Mean Fluorescence Intensity (MFI) and plotted from a single experiment using triplicate probes. Representative flow cytometry charts are shown above the graph. Statistical significance: * *p* < 0.05, ** *p* < 0.01, *** *p* < 0.001.

The VCAM-1 expression on the cells’ surface was assessed by cultivating VIC in a normal medium (NM) or HGOM for 1, 2, and 7 days. The surface expression of VCAM-1 was evaluated by flow cytometry. The results showed that VCAM-1 is constitutively expressed on some of the VIC’s surfaces. Approximately 40% of VIC grown in NM expressed VCAM-1. However, the expression of VCAM-1 was significantly enhanced by exposure of VIC to HGOM when ~80% of the cells were positive for VCAM-1 (Figure 4A).

#### 2.2.2. VCAM-1 Targeted Lipopolyplexes Are Efficiently Taken up by Osteogenic Differentiated VIC

To follow the internalization of VCAM-1 targeted lipopolyplexes by VIC, the cells were grown in NM or HGOM (5 days) and incubated for 24 and 48 h with VCAM-1 targeted lipopolyplexes containing C60-PEI/Cy3-labeled plasmid polyplexes (V-LPP/Cy3) in the absence or presence of an excess concentration of VCAM-1 binding peptide (V-BP). Then, the cells were processed for fluorescence microscopy. As shown in Figure 4B, the V-LPP/Cy3 uptake is specific and mediated mainly by VCAM-1 molecule, as attested by the reduced fluorescence when the uptake was performed in the presence of an excess V-BP. Also, the uptake is increased in the case of HGOM-activated VIC as compared with the V-LPP/Cy3 uptake by VIC exposed to NM at both investigated intervals. The quantification of lipopolyplexes uptake by VIC, expressed as fluorescence intensity signal measured by red pixels fluorescence (ImageJ software version 1.8.0) is shown in Figure 4C. The flow cytometry data obtained in the case of incubation of VIC with V-LPP/Cy3 (48 h) were in line with the fluorescence microscopy results showing that the uptake of V-LPP/Cy3 plasmid lipopolyplexes was higher for VIC exposed to HGOM compared with the uptake by VIC grown in NM (Figure 4D). The uptake is dependent on VCAM-1 expression since the addition of excess V-BP competed with V-LPP uptake and impeded the efficient internalization (Figure 4D).

### 2.3. V-LPP/shRunx2 Lipopolyplexes Downregulate Runx2 Expression in Osteoblast-Differentiated VIC

**Figure 5:**
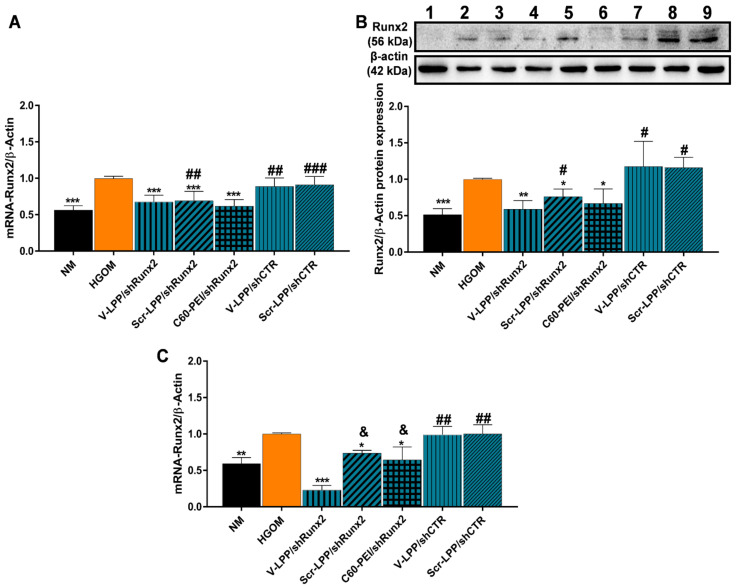
Downregulation of Runx2 expression in osteoblast-differentiated VIC. (**A**) mRNA and (**B**) protein Runx2 expression in HGOM-activated VIC for 5 days and transfected for 48 h with different type of nanocarriers. NM (lane 1); HGOM (lane 2); V-LPP/shRunx2 (lanes 3, 4); Scr-LPP/shRunx2 (lane 5); C60-PEI/shRunx2 (lane 6); V-LPP/shCTR (lanes 7, 8); Scr-LPP/shCTR (lane 9). (**C**) mRNA Runx2 level in HGOM-activated VIC subjected to double transfection in the 5th and 12th days, and measured 48 h after the second transfection (14th day). The results were normalized to β-actin and represent the mean ± S.D. of two independent experiments made in duplicate (*n* = 4) and represent fold change relative to HGOM condition (considered as 1). The samples considered statistically different were marked with * *p* < 0.05, ** *p* < 0.01, *** *p* < 0.001 when compared to HGOM; # *p* < 0.05, ## *p* < 0.01, ### *p* < 0.001 when compared to NM; & *p* <0.05 when compared to V-LPP/shRunx2.

Since we found that VCAM-1 targeted lipopolyplexes are specifically taken up by HGOM-activated VIC and delivered the plasmid cargo intracellularly, we investigated the downregulation of the Runx2 transcription factor in osteoblast-differentiated VIC by V-LPP/shRunx2. Previously, we have shown that the exposure of VIC to HGOM significantly increases the mRNA and protein levels of transcription factor Runx2, a key player in osteodifferentiation of VIC [9]. Indeed, in Figure 5 it can be observed that the exposure of VIC to HGOM for 7 and 14 days determined a significantly increased expression of Runx2 at both mRNA and protein levels; the increase was ~40% as compared to its expression in VIC grown in NM (Figure 5A,C). Quantitative Real-Time PCR experiments revealed that the transfection for 48 h of VIC, exposed previously (5 days) to HGOM, with V-LPP/shRunx2 determined a significant reduction (~35%) of Runx2 gene expression (Figure 5A). Transfection of VIC with polyplexes C60-PEI/shRunx2 determined downregulation of Runx2 gene expression by ~40% of the levels measured in HGOM-treated VIC, while the downregulation obtained using Scr-LPP/shRunx2 was ~30%. The use of the shCTR plasmid encapsulated into VCAM-1 targeted or non-targeted lipopolyplexes did not affect the level of Runx2 gene expression. Western blot assays revealed that at 48 h after transfection of five-days HGOM-activated VIC with V-LPP/shRunx2, the protein expression of Runx2 was reduced by ~40% (Figure 5B). When transfection was performed using Scr-LPP/shRunx2 and C60-PEI/shRunx2, the protein level of Runx2 was reduced by ~25% and 35%, respectively. The use of shCTR plasmid did not affect the level of Runx2 protein (Figure 5B).

At 48 h after the second transfection of HGOM-exposed VIC with V-LPP/shRunx2 (day 14 in culture), the mRNA Runx2 expression decreased significantly by ~80% (Figure 4C). The double transfection with Scr-LPP/shRunx2 determined a reduction in Runx2 gene expression by ~30%. The use of C60-PEI/shRunx2 for double transfection, induced the same decrease of Runx2 gene expression at day 14 as at day 7, after one transfection, by ~40%. No statistically significant inhibition was obtained when lipopolyplexes containing control shCTR plasmid were employed.

### 2.4. V-LPP/shRunx2 Lipopolyplexes Reduce the Expression of Osteoblast-Specific Differentiation Markers in Osteogenic-Differentiated VIC

**Figure 6:**
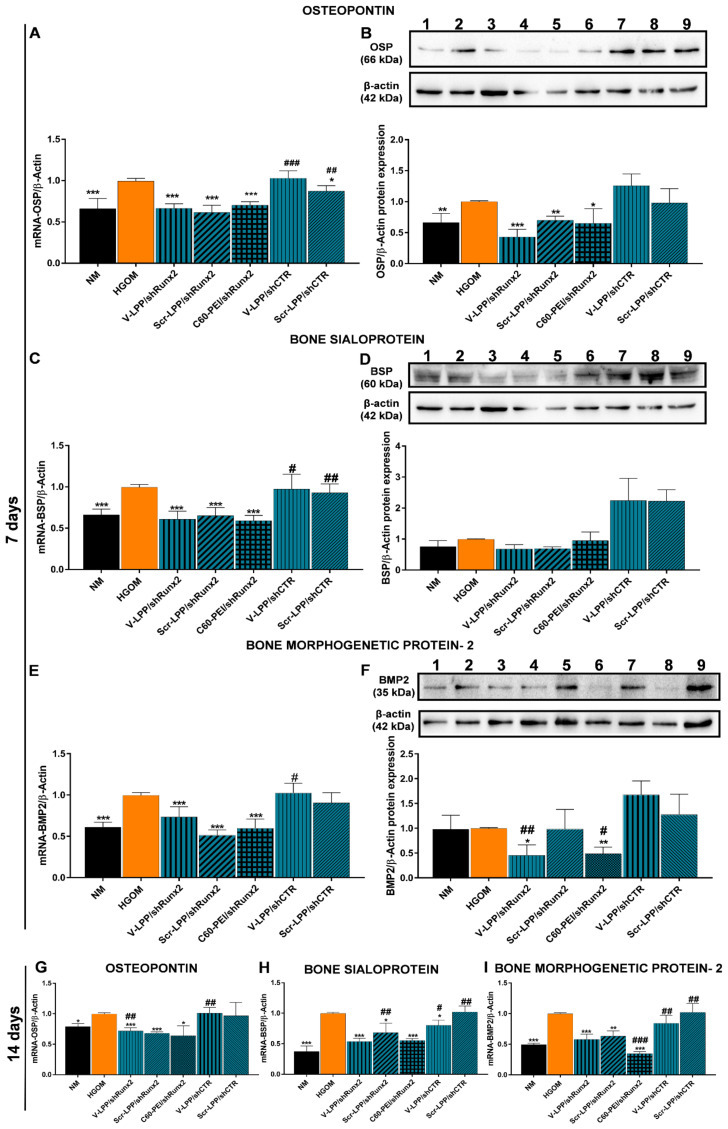
(**A**–**F**). The reduction of mRNA and protein expression of osteogenic molecules, OSP (**A**,**B**), BSP (**C**,**D**), and BMP-2 (**E**,**F**) in VIC exposed to HGOM for 7 days and transfected with a different type of nanocarriers on the fifth day. Western blots: NM (lane 1); HGOM (lane 2); V-LPP/shRunx2 (lanes 3, 4); Scr-LPP/shRunx2 (lane 5); C60-PEI/shRunx2 (lane 6); V-LPP/shCTR (lanes 7, 8); Scr-LPP/shCTR (lane 9). (**G**–**I**) The gene expression of OSP, BSP, and BMP-2 in VIC exposed to HGOM for 14 days and subjected to a second transfection on the 12th day. As controls, transfection using V-LPP/shCTR and Scr-LPP/shCTR were employed. Results, normalized to β-actin, were expressed as mean ± S.D. of two independent experiments made in duplicate (*n* = 4) and represented as fold change relative to HGOM condition (considered as 1). The samples considered statistically different were marked with * *p* < 0.05, ** *p* < 0.01, *** *p* < 0.001 when compared to HGOM, # *p* < 0.05, ## *p* < 0.01, ### *p* < 0.001 when compared to NM.

To find out whether the downregulation of the transcription factor Runx2 has a functional effect, we determined the gene and protein expression of osteogenic molecules, namely OSP, BSP, and BMP-2 in VIC exposed to HGOM and transfected with V-LPP/shRunx2 lipopolyplexes. In control experiments, exposure of VIC (7 days) to HGOM led to a significant increase (~40%) in OSP, BSP, and BMP-2 gene expression (Figure 6A,C,E). Transfection for 48 h of oVIC with V-LPP/shRunx2, Scr-LPP/shRunx2 lipopolyplexes, or C60-PEI/shRunx2 polyplexes led to a decrease in mRNA that was similar for OSP, BSP (~40%) and BMP-2 (~35%) (Figure 6A,C,E). No reduction in OSP, BSP, and BMP-2 gene expression was found in the cells transfected with the shCTR plasmid encapsulated either in VCAM-1 targeted (V-LPP/shCTR) or non-targeted (Scr-LPP/shCTR) lipopolyplexes. A higher percent reduction in OSP protein expression was determined when osteogenic-differentiated VIC (oVIC) were treated for two days with V-LPP/shRunx2 (~55%) compared with treatment with Scr-LPP/shRunx2 and C60-PEI/shRunx2 polyplexes when a decrease of ~30–35% was obtained relative to OSP protein expression in non-treated oVIC (Figure 6B). Also, the transfection with lipopolyplexes carrying the shCTR plasmid, either VCAM-1 targeted or non-targeted, did not reduce the OSP protein expression in oVIC.

Although the exposure of VIC to HGOM for 7 days determined a significant increase in BSP and BMP-2 mRNA levels, we found no significant increases of these osteogenic molecules at the protein level (Figure 6D,F), a result in line with our previous data showing an increase in BSP expression after 14 days of VIC exposure to HGOM [9]. The transfection of oVIC, on the fifth day of exposure to HGOM, with either lipopolyplexes or polyplexes does not influence the protein expression of BSP. Instead, a significant reduction in BMP-2 protein expression was determined by transfection of oVIC with V-LPP/shRunx2 and C60-PEI/shRunx2 (~50% in both cases). No effect on BMP-2 protein expression was detected in the case of oVIC transfection with Scr-LPP/shRunx2 and lipopolyplexes encapsulating the shCTR plasmid. The second transfection of oVIC with V-LPP/shRunx2, Scr-LPP/shRunx2, and C60-PEI/shRunx2 on the twelfth day of VIC exposure to HGOM maintained a decreased level of mRNA for OSP expression (~30–35%) compared with non-treated oVIC (Figure 6G). The percentages of inhibition of mRNA BSP level were ~50%, ~30%, and ~45%, whereas that of mRNA BMP-2 were reduced by ~40%, ~35%, and ~65%, 48 h after the second transfection of oVIC with V-LPP/shRunx2, Scr-LPP/shRunx2, and C60-PEI/shRunx2, respectively (Figure 6H,I). By comparison, the 2nd transfection using the control lipopolyplexes, V-LPP/shCTR and Scr-LPP/shCTR, had no effect on OSP, BSP, and BMP-2 gene expression that remained at similar levels as measured in untreated HGOM-exposed VIC at 14 days (Figure 6G–I).

### 2.5. Lipopolyplexes Are Cyto/Hemocompatible

**Figure 7:**
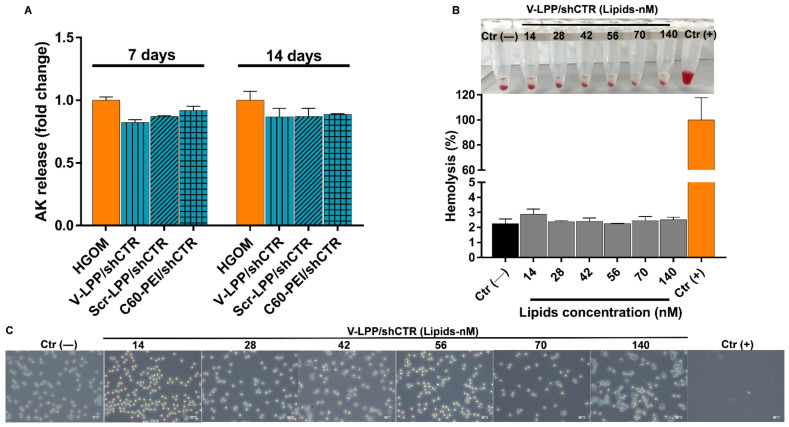
Cyto-/hemocompatibility evaluation of lipopolyplexes. (**A**) Cytotoxicity of V-LPP/shCTR lipopolyplexes measured as release of AK at 48 h after transfection performed on the 5th and 12th days. Data are presented as mean ± S.D. of one representative experiment done in three replicates. (**B**) Quantification of hemolysis in erythrocytes incubated with V-LPP/shCTR at different plasmid concentrations. The corresponding photographs of erythrocytes after their sedimentation are shown above the graph. (**C**) Evaluation of erythrocyte aggregation after incubation with lipopolyplexes by light microscopy. PBS [Ctr (−)] and 0.5% Triton X-100 [Ctr (+)]. Scale bar: 200 μm. Note that there are no significant changes in erythrocyte aggregation at any V-LPP/shCTR concentration used.

The cytotoxicity of lipopolyplexes was determined by ToxiLight assay, by measuring the adenylate kinase (AK) release at 48 h after VIC were subjected to one or two transfections with lipopolyplexes and polyplexes on the fifth and twelfth day. The data were normalized and presented as fold change relative to cells exposed to HGOM medium for 7 and 14 days, in the absence of transfection with lipopolyplexes, considered as 1. Values greater than 1 signify that a compound is toxic to cells. The data indicated that the AK release was not increased by VIC incubation with lipopolyplexes, either after a single or a double transfection. The result indicated the cytocompatibility of the developed lipopolyplexes and suggested that they can be safely used to deliver the shRNA plasmid cargo to the cells (Figure 7A).

Hemolysis and erythrocytes aggregation techniques were used to evaluate the hemocompatibility of lipopolyplexes by ex vivo incubation with erythrocytes. The hemolysis induced by V-LPP/shCTR lipopolyplexes at various lipid concentrations (14 ÷ 140 nM, corresponding to plasmid concentrations between 4.5 ÷ 45 µg/mL plasmid shRNA and 20 ÷ 200 µg/mL C60-PEI) is presented in Figure 7B. All of the V-LPP/shCTR concentrations displayed a degree of hemolysis less than 3%, even at the highest concentration (which is equivalent to a dose of 45 µg plasmid). No erythrocytes aggregation was detected, the samples behaving similarly to the negative control (Figure 7C).

## 3. Discussion

Aortic valve stenosis and calcification is an active cellular process occurring within the valve leaflet and involves pathological differentiation of the valvular cells. No pharmacological treatment to specifically slow down the progression of aortic valve calcification is currently known. VIC, the most abundant cell type in the aortic valve, plays an active role in valve calcification by acquiring an osteoblast-like phenotype in pathological conditions [20]. The Runx2 transcription factor is a key regulator of the VIC transition towards an osteoblastic phenotype as a response to various conditions such as diabetes, hyperlipidemia, or advanced age [21]. The exposure of cultured VIC to osteogenic factors, in particular dexamethasone, β-glycerophosphate, and ascorbic acid, increases the expression of Runx2 and promotes the osteodifferentiation of VIC [22]. Previously, we demonstrated that the association of osteogenic factors with high glucose concentrations has a synergic effect on Runx2 expression, accelerating the osteodifferentiation of VIC [9].

Since the shift of VIC from a fibroblast-like to an osteoblast-like phenotype (oVIC) is a critical step in the development and progression of aortic valve calcification, these cells represent a promising target for pharmacological intervention. We have previously demonstrated that Runx2 silencing in oVIC, using C60-PEI/shRunx2 polyplexes, mitigates the osteodifferentiation of VIC [9]. Here, our attempt was to develop a targeted delivery system suitable to provide shRNA sequences specific for Runx2 downregulation to the aortic valve cells once injected in vivo. We report now the design, preparation, and characterization of functional VCAM-1 targeted PEGylated lipopolyplexes encapsulating C60-PEI/shRunx2 polyplexes (V-LPP/shRunx2). The VCAM-1 was chosen as an appropriate target on the aortic VIC surface based on results demonstrating an increased VCAM-1 expression in cultured VIC activated with different stimuli or in the aortic valve of diabetic ApoE-deficient mice [17,18,23]. In this study, we demonstrated the presence of VCAM-1 on the surface of VIC isolated from non-calcified leaflets of the human aortic valve grown in NM. Exposure of the cells to HGOM leads to a significantly increased VCAM-1 expression.

The lipopolyplexes are composed of a “core” of polyplexes, made from nanoconjugates C60-PEI complexed with shRNA plasmid, surrounded by a “shell” consisting of a lipid bilayer, as revealed by TEM images. Using small-angle X-ray scattering (SAXS) would be of help to obtain a complete characterization of the structural organization of nanoparticles [24]. The V-LPP/shRunx2 were physicochemically characterized, and the results indicated a stable aqueous dispersion of particles with an average dimension of ~200 nm and a negative Zeta-potential (~−30 mV) due to the anionic lipid bilayer shell. No significant changes in the size and Zeta-potential of V-LPP/shRNA lipopolyplexes suspended in PBS were observed, whereas C60-PEI/shRNA polyplexes rapidly aggregate within 15 min. The results point out the colloidal stability of the lipopolyplexes and are in line with previous studies [11,12,13]. When systemically injected, the interaction of non-PEGylated nanoparticles with electrolytes causes their aggregation [25]. Indeed, C60-PEI/shRNA polyplexes are prone to aggregation, a fact suggested by a 2-fold increase in size when incubated in 0.9% NaC_l_, the concentration found in the bloodstream. By contrast, the PEGylated V-LPP/shRNA lipopolyplexes were not affected by the interaction with electrolytes, and no modification of their sizes occurred when exposed to NaCl concentrations equal to or higher than 0.9%. Thus, we can safely conclude that the colloidal and electrolytes stability of lipopolyplexes make them suitable for systemic in vivo administration.

We have shown a specific intracellular uptake of VCAM-1 targeted lipopolyplexes by VIC grown in either NM or HGOM, mediated by the cell adhesion molecule VCAM-1, as demonstrated by the experiments done in the presence of an excess V-BP. The increased level of VCAM-1 expression on the surface of HGOM-exposed VIC led to a higher internalization of the fluorescently-labeled plasmid cargo encapsulated into VCAM-1 targeted lipopolyplexes compared to their internalization by VIC grown in NM. The data support and extend previous studies showing VCAM-1 as a suitable target for the specific binding and internalization of nanoparticles by the endothelial cells [26,27,28,29], and emphasize the commonality of specific nanoparticles uptake by other cell types expressing VCAM-1.

Then, we questioned the direct therapeutic effect of V-LPP/shRunx2 on oVIC and found that both, Runx2 mRNA and protein levels in HGOM-exposed VIC were significantly decreased at 48 h after transfection with V-LPP/shRunx2. Two transfections were performed on the 5th and 12th days of VIC exposure to HGOM, and after the second transfection, a higher reduction of Runx2 mRNA level was achieved when V-LPP/shRunx2 were used compared to the other formulations of shRNA-Runx2 plasmid. Yet, after the first transfection, both non-targeted formulations of shRunx2 plasmid (Scr-LPP/shRunx2 and C60-PEI/shRunx2) reduce the Runx2 expression. This could indicate a non-specific cellular internalization due to a long incubation time of oVIC with nanoparticles [30]. It may be that the non-specific endocytosis of Scr-LPP/shRunx2 lipopolyplexes and C60-PEI/shRunx2 polyplexes provide the necessary amount of plasmid to reduce Runx2 expression in oVIC.

However, after the second transfection, a statistically significant difference in the reduction of Runx2 mRNA expression between targeted (V-LPP/Runx2) and non-targeted (Scr-LPP/shRunx2 and C60-PEI/shRunx2) formulations. was determined. Also, the Runx2 mRNA level is lower than that measured at seven days, after the first oVIC transfection with V-LPP/shRunx2, while, after cells transfection with Scr-LPP/shRunx2 and C60-PEI/shRunx2, the level of Runx2 mRNA was kept constant. A possible explanation of the reduced Runx2 mRNA expression obtained with targeted lipopolyplexes after the second transfection may be their higher cellular internalization, mainly by receptor-mediated endocytosis by clathrin-coated pits, as reported [26] and, also, by non-specific endocytosis compared to non-specific uptake of Scr-LPP/Runx2 and C60-PEI/shRunx2 that may become saturated. Another explanation may be the different intracellular processing of VCAM-1 targeted lipopolyplexes and non-targeted lipopolyplexes and polyplexes after internalization that affects the shRNA plasmid functionality. However, it should be noted that both Scr-LPP/shRunx2 and C60-PEI/shRunx2 produce about the same level of Runx2 expression downregulation despite their different nature. Non-targeted lipopolyplexes are PEGylated and negatively charged. Instead, C60-PEI/shRunx2 polyplexes are non-PEGylated and have a positive Zeta potential. Therefore, it is expected that different internalization mechanisms are involved in each case. Nonetheless, in our experiments, irrespective of the mechanism involved in the internalization of nanoparticles, the same effect translated into the RNAi was obtained. Further investigation, using inhibitors of endocytic pathways is needed to search into the internalization pathways involved in the process.

Importantly, the downregulation of Runx2 using VCAM-1 targeted lipopolyplexes for RNAi causes a consequent reduction in gene and protein expression of osteogenic molecules OSP, BSP, and BMP-2. The data point to the functional role of V-LPP/shRunx2 in blocking the pathological process of human aortic VIC osteodifferentiation by Runx2 silencing.

Compared to the polyplexes C60-PEI/shRunx2, which also produce downregulation of Runx2 and osteogenic molecules in cultured oVIC [9], there are two significant advantages of the newly developed VCAM-1 targeted lipopolyplexes: the suitability for in vivo administration and its potential to perform targeted delivery of shRunx2 plasmid to the diseased aortic valve.

The cytotoxicity assay, determined by the amount of released adenylate kinase (AK), indicated that the treatment with lipopolyplexes did not cause toxicity in VIC either after a single or a double transfection. This result proposes the lipopolyplexes as biocompatible materials according to International Organization for Standardization, ISO 10993-5:2009 “Biological Evaluation of Medical Devices Part 5: Tests for in Vitro Cytotoxicity, 2009”. Also, the hemocompatibility tests showed a comparable percentage of lysed erythrocytes in the case of incubation with V-LPP/shRunx2 or PBS, which was less than the threshold of 5% considered the safe hemolytic ratio for biomaterials (International Organization for Standardization (ISO) 10993-4:2017). In addition, examination of the erythrocytes after incubation with lipopolyplexes reveals a morphology similar to the negative control (PBS) at all investigated concentrations. These results illustrate the cyto- and hemo-compatibility of the developed lipopolyplexes and recommend them as safe vehicles for delivery of the cargo (shRNA plasmid) to the cells.

To preclinically validate the therapeutic effect of this RNAi vector, the results of this study justify further testing, in appropriate animal models, of this targeted nano-delivery system designed to recognize a molecular target expressed by the diseased aortic valve. We intend to follow the localization of VCAM-1 targeted lipopolyplexes in the aortic valve and the therapeutic effect of V-LPP/shRunx2 in diabetes-induced changes in aortic heart valves in a murine model of atherosclerosis, developed previously [18].

## 4. Materials and Methods

### 4.1. Reagents

The commercial sources of the main reagents and consumables used in this study were as follows: Dulbecco’s modified Eagle’s medium (DMEM), Runx2 MISSION shRNA Plasmid DNA (MISSION Runx2 shRNA plasmid DNA, cat. no. SHCLND-NM_004348 shRNA sequence specific for human Runx2, namely the clone TRCN0000013653 (sh_1) from The RNAi Consortium (TRC) Version 1 library) and 2-Oleoyl-1-palmitoyl-sn-glycero-3-phosphocholine were from SIGMA-Aldrich (Merck KgaA, Darmstadt, Germany); fetal bovine serum (FBS), penicillin and streptomycin were purchased from Gibco (ThermoFisher Scientific, Waltham, MA, USA); cell culture dishes were from TPP^®^ (Trasadingen, Switzerland); primary antibodies: mouse anti-VCAM-1 (cat no. MA5-11447), rabbit anti-Runx2 (cat no. PA1-41519), rabbit anti-osteopontin (OSP) (cat no. PA5-34579), rabbit anti-BMP-2 (cat. no. PA1-31215) and secondary antibodies: goat anti-mouse coupled with APC (cat. no. A865), donkey anti-rabbit coupled with AlexaFluor 594 (cat. no. A-21207), Quant-iT™ PicoGreen^®^ dsDNA kit and Tris (2-carboxyethyl) phosphine (TCEP), SuperSignal West Dura chemiluminescent substrate (cat. no. 34076) were from ThermoFisher Scientific (Waltham, MA, USA); antibody goat anti-bone sialoprotein (BSP) (cat. no. AF4014) was from R&D Systems (Minneapolis, MN, USA); 1,2-dioleoyl-sn-glycero-3-phospho-(1’-rac-glycerol) (sodium salt) (DOPG), 1-palmitoyl-2-oleoyl-glycero-3-phosphocholine (POPC), 1,2-dipalmitoyl-sn-glycero-3-phosphoethanolamine-*N*-(lissamine rhodamine B sulfonyl) (ammonium salt) (Rhodamine-PE), 1,2-distearoyl-sn-glycero-3-phosphoethanolamine-*N*-[Maleimide(PolyethyleneGlycol)2000] (Ammonium salt) (Mal-PEG-DSPE), 1,2-distearoyl-sn-glycero-3-phosphoethanolamine-N-[methoxy(polyethylene glycol)-2000] (ammonium salt) (PEG-DSPE) were from Avanti Polar Lipids (Alabaster, AL, USA); VCAM-1 recognition peptide and the peptide with scrambled sequence of aminoacids were from GeneCust (Dudelange, Luxembourg); SpectraPor dialysis membrane (cut-off 100–500 Da) was from Spectrum Labs (Spectrum Europe BV, Breda, The Netherlands); 100 kDa cut-off Amicon centrifugal filter columns were from Millipore (Billerica, MA, USA); ToxiLightTM Cytotoxicity BioAssay Kit was from Lonza (Basel, Switzerland); HPLC grade solvents were from Merck (Kenilworth, NJ, USA), and Cy3-labeled plasmid was purchased from Mirus Bio (Madison, WI, USA). Deionized water (18.2 MΏ/cm) was obtained in-house using a Milli-Q system from Millipore (Watford, UK).

### 4.2. Human VIC Isolation and Culture

Primary human VIC were harvested from non-calcified cusps (or portions of the cusp) of the aortic valve obtained from a patient who underwent surgical valve replacement as previously described [31]. The surgery was performed at Central Military Hospital “Dr. Carol Davila”, Cardiovascular Surgery Clinic, Bucharest, according to the Declaration of Helsinki for experiments involving human samples [32]. The patient signed the informed consent forms and his anonymity and privacy rights were respected.

VIC were cultured on 1% gelatin-coated plates and grown in DMEM 5.5 mM, supplemented with 10% fetal bovine serum, 50 μg/mL neomycin, 100 UI/mL penicillin and 100 μg/mL streptomycin (normal medium, NM), in a humidified 5% CO_2_ incubator at 37 °C. To induce the VIC activation and osteodifferentiation, the cells were exposed to a medium containing 25 mM glucose (HG) and osteogenic factors (50 µg/mL ascorbic acid, 10 mM β-glycerophosphate, 10 nM dexamethasone) (high glucose osteogenic medium, HGOM) as previously reported [9]. The Ethics Committee of the Institute of Cellular Biology and Pathology “Nicolae Simionescu” has approved the study.

### 4.3. VCAM-1 Expression in VIC

VIC were seeded in 24-well plates at a density of 50,000 cells/well. After 24 h, VIC were incubated in NM (used as control) or HGOM for 1, 2, or 7 days. Then, the cells were processed for flow cytometry analysis using a previously described protocol [33]. The quiescent and HGOM-activated VIC were detached from the culture plates, centrifuged, and resuspended in FACS buffer (0.5% PFA in PBS) followed by incubation with the primary antibody anti-VCAM-1 (1:25) for 1 h on ice. After washing in FACS buffer, cells were incubated with the secondary Allophycocyanin (APC)-conjugated goat anti-mouse IgG-antibody (1:250) for 1 h, on ice. Next, the cells were analyzed by a flow cytometer in the FL6 channel (660 nm), after excitation with the red laser (633 nm) (Gallios, Beckman Coulter, Brea, CA, USA). Data were analyzed with Kaluza Flow analysis software (v.2.1) (Beckman Coulter, Brea, CA, USA).

### 4.4. Preparation of Lipid-Enveloped C60-PEI/shRNA Polyplexes (Lipopolyplexes)

The lipopolyplexes (LPP) were prepared employing the reverse-phase evaporation method as previously described [34]. First, the C60-PEI/shRNA polyplexes were obtained as described in our previous paper [9] by mixing the C60-PEI and shRNA plasmids at a N/P ratio of 25. Each constituent was diluted separately to achieve the appropriate concentration in the same volume of 2 × HB buffer (20 mM HEPES, 10% D-glucose, pH = 7.4) and brought to 1000 µL with HB buffer (10mM HEPES, 5% D-glucose, pH = 7.4). The MISSION^®^shRNA Plasmid DNA targeting human Runx2 gene (shRunx2) (Sigma-Aldrich cat. no. SHCLND-NM_004348, clone TRCN0000013653), validated by us for silencing human Runx2 [9], and MISSION^®^ pLKO.1-puro non-Mammalian shRNA Control Plasmid DNA (shCTR), as a control plasmid, were used to obtain polyplexes at N/P = 25. The plasmids were amplified in *Escherichia coli* host strain DH5α and isolated using GenElute-Plasmid Midiprep kit (Sigma-Aldrich, Germany). The N/P ratio is the ratio of nitrogen atoms in C60-PEI to phosphorus atoms in DNA and was calculated using the nitrogen percentage resulting from elemental analysis XPS of C60-PEI (16.6% N) [9,35]. Then, 3 mM anionic DOPG diluted in 4.5 mL chloroform/methanol (2:1, *v*/*v*) was added to the preformed cationic C60-PEI/shRNA polyplexes. After incubation for 30 min at room temperature (RT), 1.5 mL of chloroform, and 1.5 mL of distilled water were added. After phase separation by centrifugation at 830× *g* for 7 min, the aqueous phase was removed, and 6.8 mM POPC, 0.1 mM Mal-PEG-DSPE, 0.1 mM PEG-DSPE (dissolved in chloroform), and 1.5 mL of HB buffer were added to the organic phase, containing inverted micelles encapsulating polyplexes, in a round bottom glass bottle, vortexed vigorously and sonicated for 1 min. The chloroform was removed by evaporation under vacuum on a rotary evaporator (Laborota 4000, Heidolph, Schwabach, Germany), at 37 °C, and lipid-coated polyplexes (lipopolyplexes) were obtained. The aqueous dispersion was extruded several times through 200 nm and 100 nm polycarbonate membranes using a hand extruder from Avanti Polar Lipids (Alabaster, AL, USA) to obtain lipopolyplexes containing shRNA-Runx2 plasmid (LPP/shRunx2) or shCTR plasmid (LPP/shCTR), uniform in sizes.

### 4.5. Coupling of VCAM-1 Binding Peptide to Lipopolyplexes

Cysteine-bearing VCAM-1 binding peptide (NH2-VHPKQHRGGSKGC-COOH) or scrambled peptide (NH2-HVKHRQPGGSKGC-COOH) were coupled to lipopolyplexes resulting in VCAM-1 targeted (V-LPP) and non-targeted LPP (Scr-LPP). First, the peptides were incubated with a reducing agent (TCEP) for 2 h at room temperature, to break the disulfide bonds. The excess TCEP was removed by dialysis overnight at 4 °C against coupling buffer (10 mM Na_2_HPO_4_, 10 mM NaH_2_PO_4_, 2 mM EDTA, 30 mM NaCl, pH = 6.7) using a dialysis membrane of 500–1000 Da. The peptides were added to LPP and mixed overnight at 4 °C to form the bonds between cysteine in the carboxy-terminal ends of peptides and maleimide in the lipid bilayers. To saturate the uncoupled maleimide groups, the LPP were mixed with 1 mM L-cysteine for 30 min at room temperature. Next, the LPP were centrifuged using Amicon centrifugal filter units of 100 kDa in order to separate the free peptide from peptide-coupled LPP. The amount of VCAM-1 recognizing peptide coupled to the surface of LPP was quantified indirectly by ultrahigh performance liquid chromatography (UHPLC), measuring the amount of peptide that remained uncoupled, as described previously [36].

The schematic representation of the preparation procedure of VCAM-1 targeted lipopolyplexes is shown in Figure 1.

### 4.6. Characterization of Lipopolyplexes

#### 4.6.1. Hydrodynamic Diameter and Zeta Potential

The size and Zeta (ζ)-potential of LPP were determined after a 1:1000 dilution in distilled water by dynamic light scattering (DLS) and electrophoretic light scattering (ELS), respectively on a Zetasizer Nano ZS (ZEN 3600, Malvern Instruments, Malvern, UK). For size, an average of 13 measurements per sample was used for an individual record and for each sample, three records were acquired. The Zeta potential was determined using a Zeta dip cell (ZEN 1002) immersed into the sample, by running three consecutive records as an average result of 13 measurements, at 5 Volts with 300 s delay between measurements. The results were analyzed with the built-in Zetasizer Software version 7.12 (Malvern Instruments, Malvern, UK).

#### 4.6.2. Negative Staining Transmission Electron Microscopy (TEM)

Five µL of the V-LPP/shRunx2 (diluted 1:10 with water) were deposited on 200 mesh formvar coated 3 mm copper grids (cat no. 2620C, SPI Supply, West Chester, PA, USA), previously treated with 0.01% poly-L-lysine for 2 min. Next, the excess liquid was blotted away, the samples were negatively stained by adding 10 µL of 1% uranyl acetate for 2 min. After the incubation period, the excess was removed by blotting and the samples were allowed to dry. The grids were analyzed with the Tecnai G2 Spirit BioTWIN Transmission Electron Microscope, equipped with a LaB6 filament (FEI Company, Thermo Fisher Scientific, Waltham, MA, USA). The TEM is equipped with a high-resolution camera FEI™ Eagle 2k CCD (FEI Company, Thermo Fisher Scientific), and was operated at an accelerating voltage of 100 kV with a beam current of 3 µA.

#### 4.6.3. Physical and Colloidal Stability of Lipopolyplexes

The physical stability of LPP was determined by measuring their dimensions and ζ-potentials at fixed time intervals (1, 2, 3, and 4 weeks), and compared to the initially measured values. The colloidal stability of LPP in phosphate buffer saline (PBS) was assessed by the DLS method over 48 min, each record being made at an interval of 8 min. Moreover, to study the effect of electrolytes on the LPP stability, the LPP/shCTR lipopolyplexes were incubated with different concentrations of sodium chloride (0.9 ÷ 5% *w*/*v*) for 1 h at 37 °C followed by particle size determinations. For comparison, the colloidal stability and electrolyte-induced flocculation of C60-PEI/shCTR polyplexes, as the core of the LPP were determined.

#### 4.6.4. Encapsulation Efficiency

The content of the encapsulated shRNA plasmids in the formed LPP was determined using the Quant-iT™ PicoGreen^®^ dsDNA kit (ThermoFischer Scientific cat. no. R11490). The fluorescent reagent stains nucleic acids for quantitating them in solution. Briefly, 40 µL of LPP were lysed in water containing 10% Triton X-100 and heparin 35 U.I. for 40 min at 37 °C. Separately, a shRNA plasmid curve of known concentrations was made in TE buffer (10 mM Tris-HCl, 1 mM EDTA, pH = 7.5), according to manufacturer instructions. The samples were incubated with 100 µL of Quant-iT PicoGreen reagent for 5 min at room temperature, protected from light. The sample fluorescence was measured using a TECAN Infinite M200Pro (Tecan Group Ltd., Männedorf, Switzerland) at excitation of 480 nm and emission of 520 nm. The encapsulation efficiency was calculated as the ratio between the amount of shRNA plasmid encapsulated into LPP and the total amount of shRNA plasmid added initially according to Equation (1). Also, the loading of shRNA plasmid into LPP was expressed as µg shRNA plasmid/µmoL lipid.

(1) $$\mathrm{Encapsulation}\;\mathrm{efficiency}\;\left(\%\right)=\frac{\mathrm{amount}\;\mathrm{of}\;\mathrm{shRNA}\;\mathrm{plasmid}\;\mathrm{loaded}\;\mathrm{into}\;\mathrm{LPP}\;}{\mathrm{total}\;\mathrm{amount}\;\mathrm{of}\;\mathrm{shRNA}\;\mathrm{plasmid}}\times 100$$

### 4.7. Determination of the Uptake of VCAM-1 Targeted Lipopolyplexes by VIC

VIC were seeded in a normal culture medium (NM) in 48-well plates, at a density of 7.000 cells/well. After 24 h the cells were incubated in HGOM medium for 5 days. Then, the cells were incubated for 24 and 48 h with VCAM-1 targeted lipopolyplexes encapsulating polyplexes formed between C60-PEI and a Cy3-labelled plasmid at N/P = 25 (V-LPP/Cy3) at a concentration of 0.3 µg DNA plasmid/well.

To investigate the specificity of VCAM-1 targeted lipopolyplexes, binding, and uptake by HGOM-exposed VIC, competitive studies, in the presence of excess VCAM-1 binding peptide (V-BP), were performed. VIC were preincubated for 10 min with a 25-fold higher concentration of V-BP as compared with the peptide coupled to the surface of lipopolyplexes, before incubation with V-LPP/Cy3. After washing with PBS, VIC were examined by fluorescence microscopy (Olympus IX81 microscope equipped with tetramethylrhodamine (TRITC) filter). Also, the cells were detached from the dishes and analyzed by flow cytometry (Gallios Flow Cytometer, Beckman Coulter, Brea, CA, USA), using blue laser excitation at 488 nm and emission at 585/42 nm in the FL2-H channel. Data were processed using Kaluza Flow analysis software (v.2.1).

### 4.8. VIC Transfection with V-LPP/shRunx2 Lipopolyplexes

VIC were seeded in a 24-well plate at a density of 35.000 cells/well and after 24 h were exposed to NM or HGOM medium. On the 5th and the 12th day after exposure to HGOM, VIC were subjected to transfections with VCAM-1 targeted or non-targeted lipopolyplexes encapsulating the shRNA plasmid with specificity for Runx2 (V-LPP/shRunx2 and Scr-LPP/shRunx2, respectively) and C60-PEI/shRunx2 polyplexes. As negative controls for RNA interference, lipopolyplexes V-LPP/shCTR, and Scr-LPP/shCTR formed with the MISSION^®^ pLKO.1-puro non-mammalian shRNA control plasmid DNA (shCTR) with no homology to known mammalian genes were used. A concentration of 1 µg shRunx2 or shCTR plasmid DNA/well was employed. At 48 h after each transfection, namely on the 7th and the 14th days, the cells were processed for Real-Time quantitative Reverse Transcription-Polymerase Chain Reaction (qRT-PCR) analysis and Western blot assay.

### 4.9. Quantitative RT-PCR

Total RNA was isolated at 48 h after incubation of VIC with lipoplexes or polyplexes using TRIzol^TM^ reagent. The RNA concentration was determined with the Spectrophotometer NanoDrop 1000 (ThermoFischer Scientific, Waltham, MA, USA). One µg of total RNA was used for the synthesis of cDNA using Moloney Murine Leukemia Virus (M-MLV) reverse transcriptase according to the producer’s protocol (Invitrogen, ThermoFischer Scientific, Waltham, MA, USA). The amplification of cDNA was performed for 42 cycles in the following optimized conditions: 2.5 mM MgCl_2_, annealing at 60 °C, and extension at 72 °C using LightCycler 480 Real-Time PCR System from Roche (Basel, Switzerland). The Runx2, OSP, BSP, and BMP-2 expressions were normalized to ACTB (β-actin) expression, and fold changes, relative to HGOM condition, were calculated using the 2-ΔΔCT method. The sequences of the primers used for analyzing the human genes of interest are given in Table 2.

### 4.10. Western Blot Assay

After exposure to HGOM medium for five days and after transfection with different lipopolyplexes (V-LPP/shRunx2, Scr-LPP/shRunx2, V-LPP/shCTR, and Scr-LPP/shCTR) and C60-PEI/shRunx2 polyplexes for 48 h, VIC were subjected to Western blot assay. The cells were washed with cold PBS and lysed in radio-immunoprecipitation assay (RIPA) buffer. The protein level in cell lysates was quantified by bicinchoninic acid assay following the manufacturer’s instructions (Sigma-Aldrich, Merck KgaA, Darmstadt, Germany). After quantifying the total protein concentration, 30 µg/lane of cell protein extracts were separated by 5–15% gradient SDS-PAGE gels. After transferring onto nitrocellulose membranes using a Trans-Blot Semi-Dry system, the blots were probed with the following appropriate primary antibodies: rabbit anti-Runx2 (1:200), rabbit anti-osteopontin (1:1000), goat anti-BSP (1:1000) and rabbit anti-BMP-2 (1:500). After washing, the blots were incubated with the appropriate secondary antibodies at RT for one hour. The membranes were, then, incubated with the chemiluminescent substrate and visualized with ImageQuant Las 4000. The densitometry of the bands was determined with ImageJ software developed at the National Institutes of Health (NIH, USA), and the results were normalized to β-actin, then calculated as fold change versus HGOM. The data were expressed as mean ± S.D. (standard deviation) of two experiments performed in duplicates.

### 4.11. Evaluation of Lipopolyplexes Cytotoxicity

Cytotoxicity was assessed using ToxiLightTM Cytotoxicity BioAssay Kit (Lonza cat. no. LT17-217), as previously reported [37]. This method measures the release of adenylate kinase (AK) from damaged cells in the culture medium. VIC were plated at a density of 50,000 cells/well in a 24-well plate and incubated with HGOM medium for 7 and 14 days, with a medium change every two days. On the 5th and 12th days, the cells were incubated with V-LPP/shCTR, Scr-LPP/shCTR, or with C60-PEI/shCTR polyplexes. At 48 h after incubation with lipopolyplexes and polyplexes (7th and 14th days in culture), the culture medium was collected for further determinations.

For quantification of the released AK, 25 μL of medium were added to a 96-well plate and incubated with 100 μL AK detection reagent for 5 min at RT. The luminescence was measured at 1 s on a Mithras LB 940 instrument (Berthold Technologies GmbH & Co. KG, Oak Ridge, TN, USA). The data were normalized to cells grown in HGOM medium, considered 1, and were expressed as mean ± S.D. (standard deviation) of two experiments made in quadruplicate.

### 4.12. Hemocompatibility Assay

The hemocompatibility of V-LPP/shCTR was investigated by measuring the hemolysis and erythrocyte aggregation induced by their incubation in the presence of lipopolyplexes as previously described [6]. Blood samples were collected from a C57BL/6J mouse (12-week-old male; Stock No: 000664, The Jackson Laboratory) in EDTA-containing tubes and centrifuged at 1000× *g* for 15 min. The resulting plasma was collected separately, and the erythrocytes pellet was diluted 1:10 in PBS, pH = 7.4, containing different concentrations of V-LPP/shCTR lipopolyplexes ranging from 14 nM to 140 nM lipids (corresponding to plasmid shCTR concentration between 4.5 and 45 µg/mL and C60-PEI concentration between 20 and 200 µg/mL) and incubated at 37 °C for 1 h. The concentrations were calculated to imitate the i.v., administration of lipopolyplexes in blood, considering that 1 mL of blood contains approximately 450 µL erythrocytes. The samples (in triplicate) were then centrifuged at 1000× *g* for 15 min to sediment intact erythrocytes and the supernatants, containing the released hemoglobin, were transferred to a flat-bottom 96-well plate and measured at 540 nm using the TECAN Infinite M200Pro instrument. The incubation of erythrocytes in PBS and in 0.5% Triton X-100 (considered 100% hemolysis), were used as negative and positive controls, respectively. The percentage of hemolysis was calculated using the following equation:

$$\%\;\mathrm{Hemolysis}=\frac{\mathrm{Absorbance}\;\mathrm{of}\;\mathrm{sample}-\mathrm{Absorbance}\;\mathrm{of}\;\mathrm{negative}\;\mathrm{control}}{\mathrm{Absorbance}\;\mathrm{of}\;\mathrm{positive}\;\mathrm{control}-\mathrm{Absorbance}\;\mathrm{of}\;\mathrm{negative}\;\mathrm{ccontrol}}\times 100$$

The sedimented erythrocytes were resuspended in PBS, placed on glass slides, and examined for aggregation using an Olympus IX81 light microscope.

### 4.13. Statistical Analysis

The results were expressed as mean ± standard deviation (S.D.) and the experiments were performed in duplicate, triplicate, or quadruplicate. Statistical analyses were performed using GraphPad Prism 7 software version 9.2.0 (332) (GraphPad Software, La Jolla, CA, USA). The statistical differences were calculated with an unpaired two-tailed *t*-test for the comparison of two groups or one-way ANOVA with multiple comparisons posthoc Tukey test for comparison of three or more groups. Statistically significance of differences: * *p* < 0.05, ** *p* < 0.01, *** *p* < 0.001.

## 5. Conclusions

To downregulate the Runx2 expression in activated VIC, we have designed and obtained targeted nanocarriers, namely lipopolyplexes consisting of VCAM-1 targeted lipid bilayer-encapsulated C60-PEI/shRunx2 polyplexes for specific delivery to oVIC. The V-LPP/shRunx2 lipopolyplexes are cyto- and hemocompatible and are specifically taken up by oVIC. These lipopolyplexes are functional in the downregulation of Runx2 expression and the subsequent significant decrease in the expression of other osteogenic molecules (OSP, BSP, BMP-2) in oVIC. The newly developed specific molecule-directed lipopolyplexes represent a promising targeted therapeutic RNAi-based strategy for CAVD by hindering the osteodifferentiation of aortic VIC exposed to pathological stimuli.

## Figures and Tables

**Table 1:** Summary of average hydrodynamic diameter, polydispersity indexes (PDI), and ζ-potential of C60-PEI/shCTR polyplexes and V-LPP/shCTR lipopolyplexes at the initial time and after storage at 4 °C for up to one month. Standard deviations correspond to independent measurements (*n* = 3).

Storage at 4 °C		Time: 0	1 Week	2 Weeks	3 Weeks	4 Weeks
C60-PEI/shCTR	Size (nm)	272 ± 20	290.9 ± 50	305.9 ± 36	363.5 ± 40	372.2 ± 47
	PDI	0.5 ± 0.02	0.5 ± 0.01	0.54 ± 0.02	0.52 ± 0.02	0.51 ± 0.03
	Zeta potential (mV)	+15 ± 2	+15 ± 3.6	+17 ± 2.8	+19 ± 3.2	+19 ± 2.4
V-LPP/shCTR	Size (nm)	188± 59	171 ± 44	165 ± 52	179 ± 47	189 ± 44
	PDI	0.21 ± 0.01	0.23 ± 0.01	0.21 ± 0.03	0.22 ± 0.03	0.22 ± 0.02
	Zeta potential (mV)	−34.2 ± 1.3	−34.4 ± 0.9	−34.2 ± 2.4	−29.4 ± 1.5	−30.2 ± 0.7

**Table 2:** Sequences of the primers for the human genes whose expression was assessed in qRT-PCR experiments.

Gene	Forward	Reverse	Amplicon (bp)
Runx2	CCGCCTCAGTGATTTAGGGC	GGGTCTGTAATCTGACTCTGTCC	132
OSP	GAAGTTTCGCAGACCTGACAT	GTATGCACCATTCAACTCCTCG	91
BSP	GAACCTCGTGGGGACAATTAC	CATCATAGCCATCGTAGCCTTG	79
BMP-2	ACTACCAGAAACGAGTGGGAA	GCATCTGTTCTCGGAAAACCT	113
ACTB	GACGAGGCCCAGAGCAAGAGAGG	CATGGCTGGGGTGTTGAAGGTCTC	231

## Data Availability

Not applicable.

## Abbreviations

AK: adenylate kinase; ALP, alkaline phosphatase; APC, Allophycocyanin; BMP-2, bone morphogenic protein-2; BSP, bone sialoprotein; C60-PEI, fullerene C60-polyethyleneimine; CAVD, calcific aortic valve disease; Cbfa1, Core-binding factor 1; CVD, cardiovascular disease; DLS, dynamic light scattering; ELS, electrophoretic light scattering; EMA, European Medicines Agency; FDA, Food and Drug Administration; HB, HEPES buffer; HGOM, culture medium containing 25 mM glucose and osteogenic factors; M-MLV, Moloney Murine Leukemia Virus; mRNA, messenger RNA; N/P, ratio of nitrogen atoms in C60-PEI to phosphorus atoms in DNA; NM, normal medium; OSP, osteopontin; oVIC, osteoblast-differentiated VIC; PBS, phosphate buffer saline; PCSK9, proprotein convertase subtilisin/kexin type 9; PDI, polydispersity index; qRT-PCR, Real-Time quantitative Reverse Transcription-Polymerase Chain Reaction; RIPA, radio-immunoprecipitation assay; RNAi, RNA interference; RT, room temperature; Runx2, Runt-related transcription factor 2; shCTR, plasmid containing scrambled shRNA sequences; shRNA, short hairpin RNA; shRunx2, short hairpin RNA-Runx2; siRNA, small interfering RNA; TAVI, transcatheter aortic valve implantation; TE, Tris-HCl with EDTA; TEM, transmission electron microscopy; UHPLC, ultrahigh performance liquid chromatography; V-BP, VCAM-1 binding peptide; VCAM-1, vascular cell adhesion molecule; VEC, valvular endothelial cells; VIC, valvular interstitial cells; V-LPP/shRunx2, lipopolyplexes functionalized with a peptide recognizing VCAM-1 and encapsulating C60-PEI/shRunx2 polyplexes.
